# The quick and the dead: a new model for the essential role of ABA accumulation in synthetic auxin herbicide mode of action

**DOI:** 10.1093/jxb/eraa178

**Published:** 2020-06-22

**Authors:** Todd A Gaines

**Affiliations:** Colorado State University, Department of Agricultural Biology, Fort Collins, CO, USA

**Keywords:** Abscisic acid, auxin, ethylene, herbicides, photosynthesis, plant hormones

## Abstract

This article comments on:

**McCauley CL, McAdam SAM, Bhide K, Thimmapuram J, Banks JA, Young BG.** 2020. Transcriptomics in *Erigeron canadensis* reveals rapid photosynthetic and hormonal responses to auxin herbicide application. Journal of Experimental Botany **71,**3701–3709.

**The synthetic auxin herbicides mimic endogenous auxin and have been used for weed control in crop, turf, and non-crop systems for decades (Busi *et al.*, 2018; Peterson *et al.*, 2016). Questions remain regarding a precise description of the mode of action of synthetic auxin herbicides, specifically about the sequence of events/processes leading to plant death. McCauley *et al.* (2020) propose that rapid up-regulation of *9-cis-epoxycarotenoid deoxygenase* (*NCED*) leading to rapid abscisic acid (ABA) synthesis and prolonged ABA accumulation followed by a general repression of photosynthesis-related transcription, and eventual leaf senescence is the primary mode of plant death for synthetic auxin herbicides.**

New synthetic auxins continue to be discovered and commercialized, such as halauxifen-methyl (Schmitzer *et al.*, 2015). The recent introduction of transgenic soybean and cotton with resistance to dicamba (Behrens *et al.*, 2007) and 2,4-D (Wright *et al.*, 2010) has focused increased attention on this mode of action, both for novel in-crop weed management as well as for the increased selection pressure for resistance in weeds and off-target movement issues (Mortensen *et al.*, 2012). Synthetic auxin herbicides interact with the auxin receptor complex and bind to Aux/IAA proteins that are transcriptional repressors of auxin-responsive genes such as *GH3* and *1-AMINOCYCLOPROPANE-1-CARBOXYLATE SYNTHASE* (*ACS*) (Grossmann, 2010; Jugulam *et al.*, 2011). Degradation of the Aux/IAA transcriptional repressors occurs following binding to the auxin receptor complex, resulting in rapid transcription of auxin-responsive genes (Box 1; Liscum and Reed, 2002).

Box 1. **Proposed pathway for synthetic auxin herbicide mode of action and plant death.**

Prior to synthetic auxin herbicide application, (1) Aux/IAA proteins repress transcription of many auxin-responsive genes, including *NCED*. (2) Application of synthetic auxin herbicide. (3) A massive dose of synthetic auxin herbicide deregulates auxin-responsive gene expression through binding to the TIR1/AFB auxin receptor, resulting in Aux/IAA ubiquitination and destruction; the auxin-responsive genes are rapidly transcribed. McCauley *et al.* (2020) propose that *NCED* is the principal auxin-responsive gene driving plant death, and its up-regulation is the critical step for the synthetic auxin herbicide mode of action. (4) *NCED* is the rate-limiting step for ABA production, and up-regulation results in rapid accumulation of ABA. (5) Transcript abundance of photosynthesis-related genes showed a general down-regulation, proposed to be an effect of ABA accumulation. (6) Plants die from loss of photosynthesis and deregulation of plant growth and development. Figure created with BioRender.

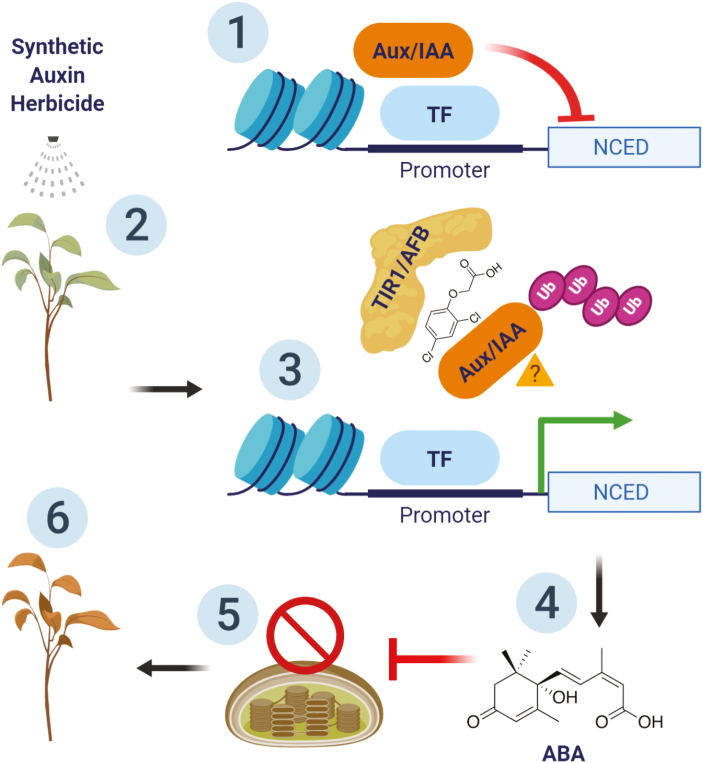

Plants have multiple Aux/IAA genes and multiple auxin-responsive genes including the rate-limiting steps for synthesis of the plant hormones ethylene (*ACS*) and ABA (*NCED*). A prevailing view has been that ethylene generation following synthetic auxin application is one of the main processes leading to plant death (Grossmann, 2010), via *ACS* up-regulation caused by synthetic auxin-mediated removal of Aux/IAA transcriptional repressor(s). Ethylene was rapidly induced following synthetic auxin herbicide application in *Galium aparine* (Hansen and Grossmann, 2000), while an ethylene perception-defective mutant in tomato did not have ABA accumulation or growth inhibition following synthetic auxin herbicide application, suggesting that ABA accumulation depended on synthesis and perception of ethylene (Hansen and Grossmann, 2000). Ethylene accumulation and increased *ACS* expression were found after dicamba treatment in sensitive *Kochia scoparia*, but neither ethylene nor *ACS* expression occurred after dicamba treatment of resistant kochia with a mutation in the Aux/IAA co-receptor gene *IAA16* (Howatt *et al.*, 2006; LeClere *et al.*, 2018; Pettinga *et al.*, 2018).

## How does a plant really die from synthetic auxin herbicides?

Enhanced *NCED* expression was detected at 1 h and 6 h after treatment with three different synthetic auxin herbicides, followed by rapid synthesis of ABA within 6 h of treatment (Box 1; McCauley *et al.*, 2020). Increased ABA synthesis was independent of loss of cell turgor due to decreased leaf water potential/stomatal closure, or increases in ethylene, both of which are potential triggers of ABA synthesis. ABA continued to increase in the days following synthetic auxin herbicide application, even more so than in plants at the point of death from drought. No difference in *ACS* expression was found at 1 h after treatment, with a slight increase in *ACS6* at 6 h after treatment. Ethylene increased only at 24 h after treatment with 2,4-D and dicamba, but not after halauxifen-methyl. Together, the consistency and immediacy of *NCED* up-regulation with subsequent ABA accumulation for all three synthetic auxin herbicides supports a primary role for the *NCED*/ABA pathway in the mode of action for plant death. Halauxifen-methyl may have some unique signalling pathways relative to 2,4-D and dicamba, which both induced ethylene at 24 h while halauxifen-methyl did not.

In addition to the up-regulation of *NCED*, at 6 h after synthetic auxin herbicide treatment, a network of genes with Gene Ontology terms related to photosynthesis was down-regulated by all three herbicides, including 52 key genes related to the Calvin cycle, light-harvesting complexes, the chloroplast electron transport chain, and chlorophyll biosynthesis (McCauley *et al.*, 2020). These trends suggest that synthetic auxin herbicides are not targeting a specific component of photosynthesis such as PSI or PSII inhibitors, but instead are causing a wholesale transcriptional down-regulation of multiple components of photosynthesis (Box 1).

## A new model for synthetic auxin herbicide mode of action reveals new research questions

Based on the findings of McCauley *et al.* (2020), synthetic auxin herbicides trigger plant death by rapidly increasing ABA through *NCED* expression, resulting in a widespread down-regulation of transcription of photosynthesis-related genes within 6 h after treatment (Box 1). Ethylene was induced by application of some, but not all, synthetic auxin herbicides, putting its role as an essential component of the mode of action into doubt.

The next step in this research is to test the hypothesis that *NCED* function is necessary for the mode of action of synthetic auxin herbicides by asking whether an *NCED* knockout in Arabidopsis is resistant to synthetic auxin herbicides. This evidence is needed to disprove the previously reported critical role of ethylene synthesis and perception in synthetic auxin herbicide mode of action (Hansen and Grossmann, 2000; Grossmann, 2010). If the function of *NCED* up-regulation leading to ABA accumulation is necessary for synthetic auxin herbicide mode of action and plant death, loss of *NCED* should make a plant insensitive to synthetic auxin herbicides. For the effect on photosynthesis, if an *NCED* gene knockout in Arabidopsis shows no down-regulation of photosynthesis genes following synthetic auxin herbicide application, then the down-regulation of photosynthesis is caused by ABA-mediated signalling. If photosynthesis is still down-regulated, then another critical signalling pathway is acting and needs to be determined.

Additional next steps would be to identify the networks of Aux/IAA proteins that repress *NCED* transcription (Box 1). This could be achieved by ChIP sequencing (ChIP-Seq) using purified protein of every Aux/IAA gene, enabling prediction of the Aux/IAA proteins that are the most likely candidates for evolved target site mutations in weeds. Weed species from different plant families could be compared for the roles of the various Aux/IAA proteins in synthetic auxin herbicide responses. For example, in *Erigeron canadensis* (Asteraceae), do similar Aux/IAA proteins regulate *NCED* as in Brassicaceae, Caryophyllales, and/or Solanaceae?

Based on the known function of the *IAA16* Gly127Asn mutation in *K. scoparia*, which changes the degron from GWPPV to NWPPV and confers resistance to dicamba (LeClere *et al.*, 2018), a testable prediction is that *IAA16* is one repressor of *NCED*. The degron of *IAA16* has also been found to cause insensitivity to both auxin and ABA when mutated from GWPPV to GWLPV (Rinaldi *et al.*, 2012). As Aux/IAA repressors of *NCED* are identified, further research can determine which are expressed during normal plant growth and development, and which are induced by high doses of both endogenous auxins and synthetic auxin herbicides. Are different Aux/IAA proteins degraded in response to different synthetic auxin herbicide chemical families, and, consequently, should we expect to find Aux/IAA mutations conferring resistance to 2,4-D in the same or different Aux/IAA genes from those for dicamba or halauxifen-methyl? Further, investigations of synthetic auxin-resistant weeds should measure *NCED* expression before and after herbicide treatment as a key marker of the mode of action. Lack of *NCED* up-regulation in resistant plants may indicate the presence of target site resistance mechanisms, such as Aux/IAA mutations. *NCED* expression after herbicide treatment should also be measured for metabolic synthetic auxin resistance mechanisms (e.g. Figueiredo *et al.*, 2018).

To enable this comprehensive understanding of synthetic auxin herbicide mode of action and allow prediction of herbicide resistance evolution across weed species, more weed genomics resources are urgently needed (Ravet *et al.*, 2018). McCauley *et al.* (2020) were able to conduct their transcriptomics research in *E. canadensis* due to the availability of a reference genome (Peng *et al.*, 2014), which they annotated themselves. In addition, transcriptomics provides hypotheses, but the development of functional tools to test these hypotheses are urgently needed to enable the study of gene function in weeds (Halfhill *et al.*, 2007; Patterson *et al.*, 2019).
